# Demographic and reproductive associations with nematode infection in a long-lived mammal

**DOI:** 10.1038/s41598-020-66075-w

**Published:** 2020-06-08

**Authors:** Carly L. Lynsdale, Nay Oo Mon, Diogo J. Franco dos Santos, Htoo Htoo Aung, U Kyaw Nyein, Win Htut, Dylan Childs, Virpi Lummaa

**Affiliations:** 1 Department of Biology, University of Turku, Turku, Finland; 2 Department of Animal Science, University of Veterinary Science, Yezin, Myanmar; 3 Department of Animal and Plant Sciences, University of Sheffield, Sheffield, UK; 4 Myanma Timber Enterprise, Ministry of Natural Resources and Environmental Conservation, Yangon, Myanmar

**Keywords:** Behavioural ecology, Evolutionary ecology, Population dynamics

## Abstract

Infection by macroparasites, such as nematodes, varies within vertebrate host systems; elevated infection is commonly observed in juveniles and males, and, for females, with different reproductive states. However, while such patterns are widely recognized in short-lived model systems, how they apply to long-lived hosts is comparatively understudied. Here, we investigated how infection varies with host age, sex, and female reproduction in a semi-captive population of individually marked Asian elephants *Elephas maximus*. We carried out 1,977 faecal egg counts (FECs) across five years to estimate nematode loads for 324 hosts. Infection patterns followed an established age-infection curve, whereby calves (5 years) exhibited the highest FECs and adults (45 years) the lowest. However, males and females had similar FECs across their long lifespan, despite distinct differences in life-history strategy and clear sexual dimorphism. Additionally, although mothers invest two years in pregnancy and a further three to five years into lactation, nematode load did not vary with four different measures of female reproduction. Our results provide a much-needed insight into the host-parasite dynamics of a long-lived host; determining host-specific associations with infection in such systems is important for broadening our knowledge of parasite ecology and provides practical applications for wildlife medicine and management.

## Introduction

One of the most recurrent patterns in parasite ecology is that parasites are aggregated within host populations, meaning that the majority of parasites are observed within the minority of hosts^1–3^. Indeed, this is so widely supported that it has been proposed as a general law in parasite ecology^4^. In natural populations, individual hosts often vary in their observable measures of infection. Such host heterogeneity in infection arises from a broad suite of factors, both external to the host and pertaining to host-specific ‘intrinsic’ traits. Particularly, associations between parasite infection and host age, sex and reproduction have been a key focus within the literature, and have been established within a range of vertebrate hosts including primates^5^, sheep *Ovis spp*^6–8^, rodents^9–11^, and birds^12,13^.

Host age has a pivotal role in influencing patterns of macroparasite infection, but the patterns observed depend on the host system, occupant parasite(s), and measure of infection used (e.g. faecal egg counts, worm counts, prevalence etc). When considering abundances of harboured parasites, or parasite loads, early theoretical work outlined three hypothetical age-intensity curves; linear increases (type I), increases towards an asymptote (type II), or an initial increase to a maxima before declining (type III)^3,14^. Studies in vertebrates have described peaks in parasite loads at specific ages, e.g.^15^, which has been attributed to changes in immune function across lifespan^16^. Unlike innate immunity, which is established during prenatal development, adaptive (or acquired) immunity is accumulated following birth and over an individual’s lifetime, through exposure to external pathogens and experience of infection. This has been extensively detailed in humans^17^; juveniles, who typically are less likely to have encountered pathogens and disease, are less able to effectively respond to immune threats due to their immature adaptive immunity. Adults by comparison may have been challenged by, and overcome, infection multiple times over their longer lifetimes, and so are generally less vulnerable. However, whilst peaks in infection are widely observed in a number of host taxa, different age-specific infection patterns have been reported within some host systems. In primates for example, infection has both negative^18^ and positive^19^ linear correlations with host age, as well as non-linear distributions where infection peaks at certain ages^5^.

Sex-biased parasitism is widely reported in the literature, and males are often more heavily parasitized than females^3^. This is thought to be driven in part by different selection pressures acting on males and females in life-history strategy, and differential resource allocation trade-offs between immunity and reproduction^20^. Males, limited in fecundity by the number of mates fertilized^21^, often increase their fitness through investment in risky behaviours or showy secondary sexual characteristics at the expense of immunity^12^. Conversely, females are restricted by the number of progeny produced^21^, and thus may benefit from increased longevity and higher investment in offspring following reproduction^22^. Therefore selection for investment in reproduction at the expense of immunity, resistance to infection, and longevity may be more pronounced in males than females^23^. Additionally, sex-specific variation in parasitism may be further reinforced by differential endocrine influences on male and female immunity, i.e. by oestrogen promoting immune function and testosterone acting as an immunosuppressor and as a handicap, at the same time as promoting secondary sexual characteristics^24,25^.

Associations between infection and host reproduction are complex; from one perspective preferential resource investment in immunity over reproduction may lead to impaired host reproduction through, for example, reduced fecundity or decreased reproductive output (e.g. lifetime offspring production)^26,27^. In extreme cases, infection may lead to complete reproductive restriction in the form of castration^28^. From the reverse viewpoint, host investment in reproduction is also costly and resource allocation trade-offs may favour reproductive effort at the expense of immune function and parasitic resistance. Within mammals particularly, females bear high metabolic and physiological costs following reproduction, for example during pregnancy, with parturition and throughout lactation, and from other post-partum care^29^. It follows that variation in host reproductive state may influence the success rate of establishing parasite fauna and allow infection to expand under reduced host immunity, which is concordant with empirical evidence of higher parasite loads in mothers and pregnant females than in non-reproductive females^30,31^. Indeed, this is a longstanding finding in veterinary and agricultural studies, which outline periparturient lapses in resistance, which is relaxed following parturition, even in lines bred for genetic resistance^32^.

While parasite aggregation and host heterogeneity are long-standing concepts in parasite ecology, comparatively more of the evidence is based on cross-sectional observations, or from laboratory or short-lived host systems. Although some headway has been made in understanding infection in long-lived, free-ranging systems^19,33–36^ this skew in the literature is a significant oversight, highlighting a noticeable gap in our knowledge of how host-parasite dynamics operate in long-lived systems over longitudinal time-frames. In comparison to shorter-lived systems, long-lived hosts present a different ecological framework for parasites to function within; hosts persist over longer periods, providing a more stable environment for parasite establishment and enabling chronic infections, which may span years rather than months or days. Consequently, different selective pressures may act on long-lived hosts and their occupant parasites in and those of shorter-lived host systems, potentially leading to variation in parasite abundance and diversity across different host lifetimes as a result. However, results from current studies on host life-history and infection levels are mixed^37^. Additionally, the fitness and survival consequences of infection may vary between hosts suffering from shorter- or longer-term parasitism. For example in chronic infections hosts may experience morbidity or slow declines in health^38^, as well as extended endocrine disruption^39^, potentially as a result of slow parasite population growth over prolonged time periods. Conversely in acute infections parasite numbers may expand rapidly, leading to rapid loss in host condition and, in severe cases, mortality, e.g.^38^. Therefore the selection pressures acting on both host and parasites in chronic infections may be different to those across shorter-term infections, which in turn may lead to evolutionary outcomes^37^, for example investment in tolerance, resistance, or both as a host defences^38^.

It is also important to quantify infection at the individual level, in order to understand how infection shapes individual fitness across different host demographics, and subsequently how infection may regulate host populations. For example, although host health and survival are expected to decrease with increasing parasite loads, this relationship is not always linear^3^. Considering that macroparasites are aggregated across hosts, few, specific hosts within a population should experience bigger impacts on their fitness as a direct consequence of heavy infection. The consequences of elevated infection for these specific individuals may be felt at the population level, especially if certain hosts groups regularly carry the highest parasite loads. For example, instances of extreme infection leading to host mortality, the selective disappearance of key demographics, such as juveniles, may lead to disruption of population dynamics^40^. However, many factors pertaining to the host, parasite and external environment should be considered to disentangle the population-scale effects of infection from other confounding biological processes^41^.

Here, we investigated host-specific patterns of gastro-intestinal nematode infection, namely strongyles and *Strongyloides spp*., in a rarely studied and extremely long-lived host system, the Asian elephant *Elephas maximus*. First, we established whether nematodes are aggregated within the elephant population. We next determined whether variation in infection is associated with heterogeneity in several key host traits: age, sex, and four measures of female reproduction: reproduction (offspring production) within a female’s lifetime prior to sampling, reproduction within five years prior to sampling, reproductive state (pregnant/not pregnant) and lifetime reproductive success (number of calves produced prior to sampling). We carried out nematode faecal egg counts (FECs, an estimate of parasite load) on individually-marked animals over five years, accounting for seasonal and temporal variation, to determine how longitudinally measured nematode loads varied across ages, between sexes, and between reproductive (mothers and pregnant females) and non-reproductive females, and with female lifetime reproductive output. Specifically, we predicted higher FECs in juveniles and elderly adults rather than adults in the middle of their lifespan, and in males in comparison to females. Additionally, we expected that females that invested more in reproduction, i.e. mothers and pregnant females, would exhibit higher FECs relative to non-reproductive females, and that FECs would be higher for females that had produced more offspring over their lifetime and thus experienced higher overall costs of reproduction. Finally, we established whether nematode load is repeatable within individual hosts by assessing the variance in FECs attributed to individual elephant ID.

The working Asian elephant population of Myanmar offers a distinct opportunity to address these questions: the semi-captive elephants exhibit similar vital rates and nocturnal behaviours to wild conspecifics^42,43^, but are individually recognizable, meaning that we can regularly collect longitudinal repeated measures from elephants of known age, sex, and reproductive history. Gastro-intestinal (GI) nematodes are of the most abundant and damaging parasites of Asian elephants^44^. GI nematodes have been observed in recent years in the working population^45^, and the elephants are routinely treated for infection (see Methods for further details). Historically, GI nematodes have been regarded as a health threat for over a century, and are an important driver of mortality^46^, in our host system. However, despite their recognition as a cause for concern, patterns of aggregation and repeatability in nematode infection in individual elephants has not yet been explored, nor how such aggregation patterns vary with host demography.

## Results

Results are adjusted for differences in anthelmintic treatment history, collection season, and work camp location. All means and model estimates are given as ± standard error, with goodness of fit confirmed for model analysis through residual diagnostic checks. We observed strongyle- (Nematoda; Strongylidae) and *Strongyloides-* (Nematoda; Strongyloididae) type eggs (see Supplementary Fig. S1) in different developmental stages (morulated, embryonated and larvated). Ova were approximately 60–90 μm × 35–50 μm, and were typically clear, thin-shelled, and oval/ellipsoidal in shape.

### Aggregation of nematodes across hosts

Nematode loads varied between individual elephant hosts, with FEC ranging from zero to 4060 epg. Mean FEC was 176 epg ±10. Approximately one quarter of all hosts sampled (n = 85), experienced loads of at least 500 epg, considered ‘high’ shedders in equine guidelines^47^, or more at some point during the study. The variance-to-mean ratio of number of nematode eggs for the population was >1, suggesting that there was significant heterogeneity between hosts in infection, and that nematodes were aggregated within the elephant population (Supplementary Fig. S2). We calculated the parameter of aggregation, κ, as κ = 0.301, indicating that nematode eggs were highly aggregated across hosts within the elephant population. Furthermore the variance-to-mean ratio was consistently >1 across collection years, and although κ varied year-to-year it was above 0.4 for 4/5 years during the total study period (2013: 0.526; 2014: 0.211; 2015: 0.458; 2016: 0.495; and 2017: 0.614). This suggests that nematodes were consistently aggregated within the host system over time.

### Variation in nematode load with host age and sex

**Figure 1:**
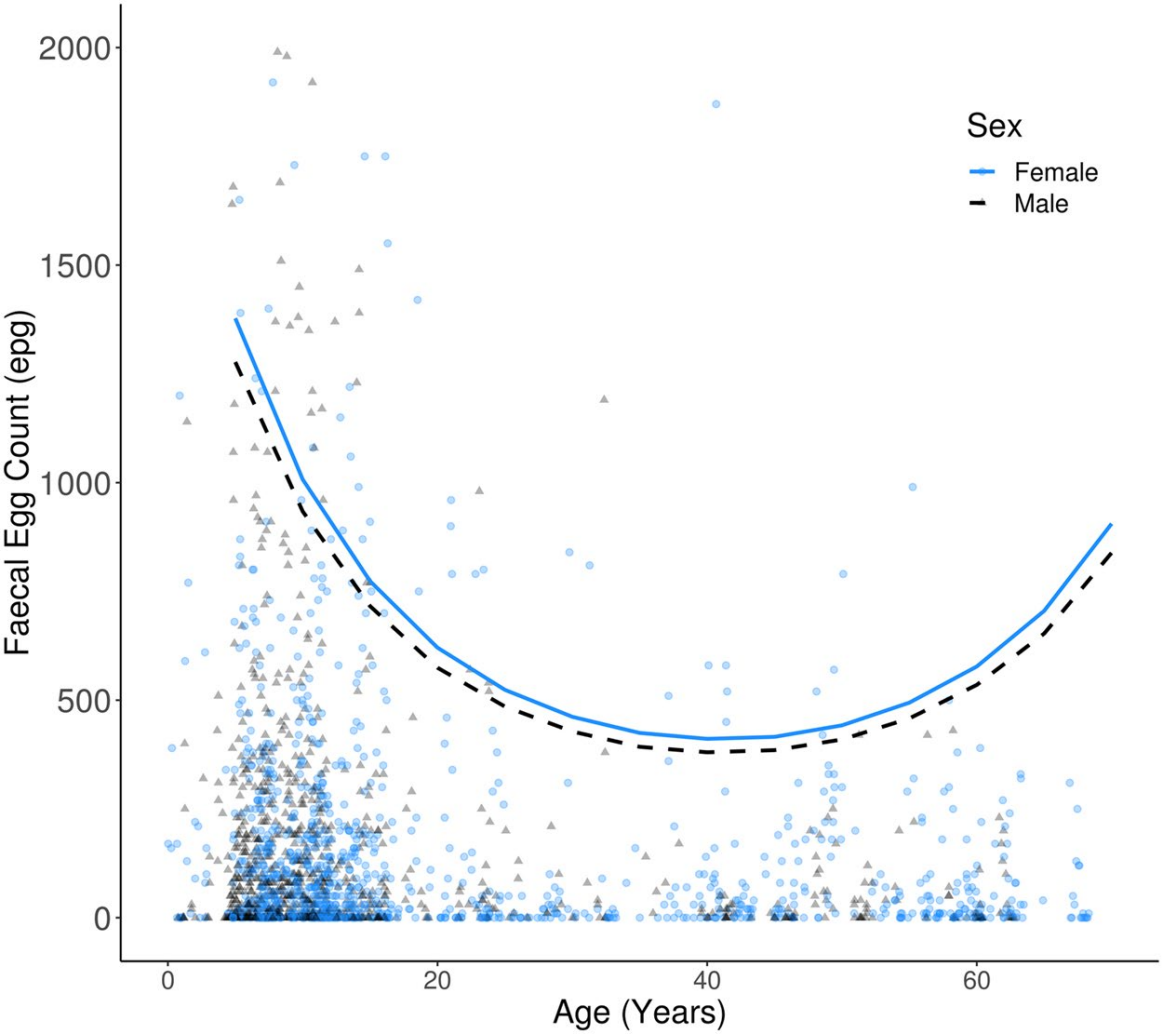
Raw (points) and mean model predicted (lines) values for faecal egg counts (epg) across elephant ages and between sexes, totalling 1,977 data points from 324 elephants. Predicted values were generated by averaging across camp, treatment, collection year and elephant ID using the predictInterval function from the *merTools*^80^ package in *R*. We specified predictions to a 95% confidence level and for 1000 simulations per observation. Predicted values were calculated using our final model structure and with an expanded dataset (using expand.grid on our original dataset of 1,977 FECs) which combined all levels for each categorical model predictor and five year increments in elephant age from 5–70 years old. Each expanded data value was fitted to 365 days since last anthelmintic treatment. Plotted data is limited to FECs of 2000 epg and under for elephants under 70 years of age.

Nematode load varied significantly with elephant age, and with a quadratic age term best explaining associations with FEC (linear age: Χ^2^ = 34.18, *P* < 0.001; age^2^: Χ^2^ = 18.49, *P* < 0.001; age^3^: Χ^2^ = 0.02, *P* = 0.899). The youngest calves suffered the highest infection (mean model predicted FEC for calves aged five years = 1336 ± 14 epg). Nematode loads then declined with age until 45 years when predicted FECs were lowest (403 ± 4 epg), after which FECs increased until the end of lifespan (683 ± 7 epg at 65 years). FECs remained relatively stable between ages 35 to 55 years, see Fig. 1.

Contrary to our expectation, we found no significant difference between sexes in overall nematode load (Χ^2^ = 0.48, *P* = 0.487). Instead, males and females experienced similar loads on average (181 ± 11 epg for males and 172 ± 9 epg for females, model estimate = −0.077 ± 0.112, Fig. 1, Table 1). We also found no evidence of an interaction between sex and age, associated with nematode burden (sex*age: Χ^2^ = 0.57, *P* = 0.456; sex*age^2^: Χ^2^ = 3.75, *P* = 0.153), showing that age-related changes in infection followed comparable trajectories in both sexes.

**Table 1:** Effect estimates on the log scale for predictors of faecal egg counts (FECs) in Asian elephants.

Model Coefficient	Estimate ± SE	*Z*	*P*
Intercept	2.244 ± 0.267	8.418	<0.001
Age (linear)	−13.927 ± 2.353	−5.918	<0.001
Age (quadratic)	9.981 ± 2.310	4.324	<0.001
Sex (males)	−0.077 ± 0.112	−0.681	0.496
Days since treatment	0.071 ± 0.004	15.888	<0.001
Camp (Kawlin)	−0.903 ± 0.157	−5.757	<0.001
Camp (West Katha)	−1.394 ± 0.171	−8.166	<0.001
**Random Effect Coefficient**	**Variance**	***Std. Dev****.*	
ID	0.368	0.607	
Collection year	0.229	0.479	

Model output from the final demographic model framework, with a negative binomial error structure and log link function. The intercept corresponds to FECs for elephants at age zero, for female elephants, with zero days since last treatment, and from East Katha. Individual elephant identification number and year of sample collection were included in the models as random effects. Models were fitted to 1977 observations from 324 elephants, with samples collected across five years.

### Variation in nematode load with female reproduction

**Figure 2:**
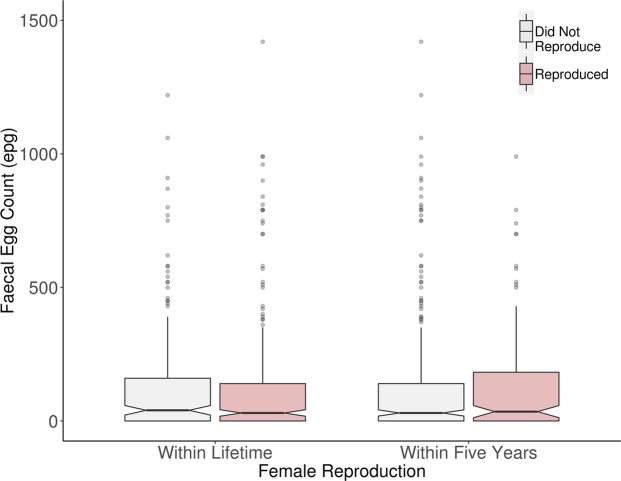
Faecal egg counts (epg) were similar between all 133 females, regardless of individual investment in reproduction. Females which invested in reproduction, either in the long-term (over their entire lifetimes to the date of sampling) or recently (within five years of sampling) are shown in red, and those which did not reproduce within the corresponding time frames in grey. The top and bottom horizontal black lines of boxplots represent the first and third quartiles of the data range respectively, the medians are shown by middle horizontal lines, and the data range shown by vertical lines, with outliers plotted as points. The notches of each boxplot are approximate 95% confidence intervals of medians. Plotted data is limited to FECs under 1500 epg.

Nematode loads for reproductive aged females (13 years and older) ranged from zero to – 4060 epg, with 23 females experiencing relatively high measures of infection (loads of 500 epg or greater). Contrary to our expectation, nematode loads did not vary with any measure of investment in female reproduction. Regarding long-term investment and lifetime reproductive output, FECs were similar between non-reproductives and mothers which had produced a calf at some point within their lifetime before sampling (Χ^2^ = 1.43, *P* = 0.232), and did not vary with the number of offspring produced before sampling (Χ^2^ = 1.41, *P* = 0.235). Regarding short-term investment in reproduction, FECs remained similar for females irrespective of whether they had reproduced within five years of sampling (Χ^2^ = 0.35, *P* = 0.556), or whether they were pregnant or not at the time of sampling (Χ^2^ = 2.18, *P* = 0.140). In fact, although not significantly different, FECs were higher on average for nulliparous females who had never reproduced at the time of sampling (148 ± 25 epg) compared to mothers (143 ± 17 epg Fig. 2). In contrast, FECs were on average higher for pregnant females (289 ± 74 epg), as well as mothers who had recently reproduced (150 ± 22 epg), than non-reproductives (non-pregnant females 130 ± 21 epg; females who had not recently reproduced within five years of sampling 142 ± 18 epg) but these differences did not reach statistical significance.

### Individual repeatability of infection

Nematode loads were somewhat repeatable within hosts that had been sampled multiple times over the study period. Approximately 8–18% of variation in FECs was explained by individual ID (*R* = 0.13 ± 0.025 [0.084, 0.182], *P* < *0.001*).

## Discussion

Macroparasites, such as nematodes, are typically aggregated across hosts. Aggregation of parasites may arise, in part, due to heterogeneity in intrinsic host characteristics^3^. Previous research has outlined associations between heterogeneity in infection and host age, sex, and reproduction, but this remains relatively understudied in long-lived and non-laboratory host systems. Here, we determined how infection by gastro-intestinal nematodes was associated with individual age, sex, and female reproduction, in a long-lived host living in natural forest habitat. Understanding how intrinsic traits vary with infection in nature not only broadens our knowledge of how parasite dynamics operate in different host systems, but also provides practical applications to wildlife medicine and welfare, e.g. through informing targeted treatment schedules of managed systems.

We found that nematodes were aggregated, with high loads (FECs > 500 epg) observed in relatively few elephants. Furthermore, we found that infection was repeatable within individuals, with hosts incurring somewhat repeatable FECs, suggesting that the same few hosts may consistently harbour high loads. Nematode load varied significantly with host age, with FECs highest in juveniles, but surprisingly we found no evidence to suggest differences in infection between males and females at any age. Additionally, mothers and non-reproductive females had similar FECs, suggesting that nematode loads were comparable between females irrespective of individual investment in reproduction against expectations. It is important to identify those demographics that exhibit higher estimates, and therefore may be more at risk, of nematode infection.

In vertebrates, ageing is coupled initially with the development, and later the senescence, of immunity. As predicted, we found higher nematode faecal egg counts in the youngest calves under weaning age (five years). It is generally accepted that acquired immunity is underdeveloped in young individuals, which can lead to increased parasite loads and prevalence. In other comparably long-lived hosts, such as humans, increased parasite loads in children are commonly observed within endemically infected communities; In a review of literature assessing major helminth species infecting humans, Galvani^48^ found that mean peak parasite load is observed at 10 ± 4 years on average. This suggests that adults and those nearing maturity have built effective protective immunity to prevent heavy infestations. Concordantly, the lowest predicted nematode load in our study was at 45 years old, just over the mid-point in Asian elephant potential lifespan. However, loads then increased in elephants past 45 years, which may suggest resurgences in susceptibility with the onset of old age. Evidence from other long-lived species such as roe deer (*Capreolus capreolus)*^49^ and Soay sheep (*Ovis aries*)^50^ suggests that immunosenescence may occur as a mechanism of age-related physiological change. However, while the apparent increases in FEC in elderly elephants are concordant with deterioration in parasite resistance due to immunosenescence, specific data on immune function and other measures of infection would be needed to confirm this.

Our results outline a distinct comparison to other long-lived taxa such as humans, but contrast with other studies on Asian and African elephants (*Loxodonta africana)*, which mostly report either no association between age and parasite infection^51,52^ (but see Parker *et al*.^53^ for evidence of increased juvenile infection in African elephants) or both increasing and decreasing faecal egg counts in different social groupings^54^ with increasing age. However, these studies are constrained: ages were estimated^52,54^ or restricted in range (two – 36 years^52^, and seven – 21 years^53^). Furthermore, individuals were identified by morphology rather than ID number^51,52,54^, allowing for misidentification, which may inflate individual variance in estimated parasite loads or increase sample sizes from pseudoreplication.

In contrast, our study was carried out on a large sample of individually-marked animals over five years, with known age, sex and reproductive history and representing the entire range of lifespans experienced by elephants in the wild (<1 year to >72 years of age). However, also our study had limitations. We estimated burdens using faecal egg counts via a special modification of the McMaster method. Faecal egg counts are widely implemented in both veterinary medicine and disease ecology to quantify infection for a range of gastro-intestinal parasites and host systems. However it should be noted that this method cannot directly indicate ultimate parasite burden and has a number of caveats, which are outlined in the methods. Additionally we could not experimentally induce or increase infection in our study, nor could we include an untreated control cohort. As such our study was correlative and investigated associations between estimates of nematode infection and a number of host-specific traits. Yet, interestingly, our results of heightened infection in juveniles and elderly hosts mirrors patterns of increased parasite-associated mortality at similar ages within the same population^46^. The increases in both current levels of infection and the relative parasite-associated mortality risk in these age groups suggests that not only may these individuals be more likely to have established burdens, but they are also potentially more likely to suffer heightened consequences to health and condition because of them, which is worthy of further exploration in future studies.

Sex-biased parasitism arises via a number of ecological and physiological mechanisms^55^, as host sex presents an obvious dichotomy in many taxa, where males and females in many species differ in morphology, endocrinology, behaviour, and life-history strategy. Heterogeneity in such traits may lead to differences in exposure and transmission^56^, immunity^57^, and influences selection pressures acting on males and females^58^. Generally, male vertebrates, often being bigger, bolder, and investing less in longevity and immunity than females, are commonly reported as suffering from elevated parasitism^3^. As such, we expected higher FECs in bulls. Yet we found no evidence to suggest any parasitism bias in either sex, with only negligible differences in nematode FECs between male and female elephant of any age. However, male-biased parasitism is by no means universal with some studies showing higher parasite loads in females^11^, including in African elephants under 21 years old^53^. Fewer studies still have observed a complete lack of sex-based bias in parasite load (e.g. as with the big brown bat *Eptesicus fuscus*^59^). We suggest that other factors may instead more strongly influence nematode aggregation in our study population, which may override other heterogeneity in host susceptibility between elephant sexes, including potentially group structure and differences in parasite composition. Previous literature shows that increased social proximity^56^ can lead to increased infection through higher rates and routes of transmission. In nature elephants live in matriarchal herds of females and their juvenile offspring; males nearing puberty leave herds or are forced out, afterwards being nomadic and living predominantly solitary existences^60^. While the working elephants are free-roaming when released at night, close proximity of mixed-sex working groups during daylight hours allows for close proximity of mixed ages and sexes, differing from their natural social organization. While the effect of proximity on parasite infection remains untested in the working elephants, their group structure may allow for increased transmission between sexes, which would normally be reduced in nature, and may mask inherent differences in susceptibility. Additionally, our results add to a complex picture of male infection in this population: while in our study we found males do not carry higher nematode loads than females, as estimated by FECs, results from previous work has shown that they are nevertheless more likely to die from their occupant parasite burdens compared to females^46^. While underlying mechanisms behind this remain unclear, one suggestion is that male parasite composition may differ from that of females, and males may harbour more pathogenic parasite species, incurring more severe impacts on health, condition and survival.

In many mammal species, females bear the brunt of reproduction through high investment of physiological resources, both during pregnancy and following parturition. For spotted hyaenas *Crocuta crocuta*^61^, and Soay sheep^62^, more fecund females suffer higher parasite loads suggesting that resources are invested in reproduction at the expense of immune function. In humans, the increased parasite infection in fecund females is well documented, with medicated regimes designed specifically for pregnant and nursing women living in high-risk regions^63^. Elephant females invest heavily into reproduction, having the longest gestation period of all mammals and lactation that can last three to five years^64^. It is therefore surprising that mothers, pregnant females and non-reproductive females had similar FECs, and that estimated nematode loads did not significantly vary with increasing lifetime reproductive output. Intriguingly, elephant mothers in our population experience a significantly reduced risk of parasite-associated mortality than non- reproductive females^46^. One possibility is that in an extremely long-lived mammal such as Asian elephants, only better quality females invest more heavily in reproduction, thus minimizing the negative impacts of infection on health and survival: for example, a previous study found that taller Asian elephant females were more likely to have reproduced by a given age, but such effects diminished with age, suggesting there may be a size threshold to reproduction, which is especially important in young females^65^. Females may also postpone reproduction if faced with, or recovering from, a high health threat such as overwhelming parasite loads or disease requiring increased investment in immunity, as modelled by Smith *et al*.^66^, favouring survival over reproduction, which may be a particularly effective strategy for a long-lived host. This may be particularly relevant for systems such as ours, where increased infection occurs in younger ages, which may subsequently impacts host fecundity in later life. The mechanisms underlying our result therefore remain elusive and present a wide scope of opportunity for further study, particularly investigating the later life consequences of heightened juvenile infection.

Here, we provide a rare insight into the host-parasite dynamics of a long-lived host system, using longitudinal, repeated measures from known individuals to determine individual-variation in nematode load in this correlative study. We find agreement with previous mammalian parasite studies, which typically report high juvenile infection and increased loads at later life-stages. In contrast, we find no evidence for sex-biased parasitism or for trade-offs between immunity and multiple measures of female reproduction. By determining which host demographic groups suffer higher or lower nematode loads, we provide an insight as to which hosts may have elevated or decreased infection respectively. Our study contributes to a small but growing body of literature detailing parasite infection in large, long-lived vertebrate hosts, experiencing natural seasonal and environmental variation. We conclude that more studies on such systems are needed in order to improve our understanding of how host-parasite dynamics operate across different ecological contexts.

## Methods

### The Myanmar timber elephant population

Myanmar houses the largest captive population of Asian elephants in the world (>5,000), over half of which (~2,700) are working timber elephants^64^ owned and managed by the Myanma Timber Enterprise (MTE). The working elephants are semi-captive: they are managed during the day but released at night, when they can roam unsupervised in the surrounding forest, and forage, interact, and mate with other semi-captive and wild conspecifics. During working hours, the elephants are organized into mixed-sex groups of approximately five - six adults, situated in forest camps, across the country. Workload is designated by hauling capacity and age. Calves do not work and remain with their mothers until approximately four to five years of age when they are weaned and undergo taming. Pregnant females are rested from approximately 11 months through gestation until a year following parturition^43^. Mothers of calves older than two years are given light work, and calves suckle as needed. All elephants are rested during the hottest, driest months (March – May), with the working season beginning again at the start of the monsoon (June – October) and extending throughout the cold season (November – February)^64^.

To facilitate working capability and wellbeing, the elephants are regularly checked by trained veterinarians and appointed a human caretaker, or mahout. Mahouts work with the elephants during the day while veterinarians are charged with their basic, restorative care, e.g. treating wounds and other work-sustained injuries. Anthelmintic drugs (namely Ivermectin and Albendazole^67^) were introduced in the 1990s and are administered by veterinarians, but this practice is intermittently enforced across working camps throughout the country. In camps where anthelmintics are given, a state-regulated blanket treatment is rolled out across all elephants approximately twice a year, irrespective of nematode load. Treatment is administered either subcutaneously at a dosage of 1 ml/100 kg body weight, or orally at a dosage of 10 mg/100 kg body weight for Ivermectin and 750 mg/100 kg body weight for Albendazole (personal communication, Dr Htoo Htoo Aung), following recommended doses for equines. A baseline study suggests that treatment is effective in reducing strongyle burdens within 90 days of administration^68^. Exact dates of anthelmintic treatment are recorded onsite on the day of administration in elephant log-books.

To investigate variation in nematode load, we carried out 1,977 FECs from 324 elephant hosts over a five year period (November 2013 – April 2017). We collected samples from three sites across Sagaing region, northern Myanmar: Kawlin (1,372 samples, 141 elephants), West Katha (353 samples, 93 elephants), and East Katha (252 samples, 90 elephants). Host age (at the time of sample collection) ranged from seven days to >72 years, nearly the full scope of Asian elephant lifespan^69^. Our study population had a relatively even sex ratio, with a slight female bias of 188 females (1073 samples) to 136 males (904 samples), and the age distribution of the sexes was similar (females < 1 year to >72 years, males: <1 year to >62 years). The majority of elephant hosts were captive born (252) as opposed to being caught from the wild, with six elephants of unknown origin. We obtained repeated samples from 232 hosts (approx. 95% of total data, corresponding to approx. 72% of all elephant hosts). All hosts had been dewormed during their lifetime, with time since last treatment varying between hosts (1–705 days at the time of sampling; mean time since treatment per host 7–510 days; population mean 125 days). For approximately 77% of measures (n = 1514/1977) treatment had occurred within the study period (29.11.13–03.04.17).

### Demographic and reproductive data collection

Each timber elephant is identifiable by a unique, four-digit identification number marked on their haunches^43^, and individual log-books are also assigned to each animal. We obtained information on elephant life-history and health data, and, for females, calving events, all of which is recorded in the log-books of each study animal throughout their lifetime^64^, and would otherwise be challenging to obtain for such a long-lived mammal. Dates of birth are exact for captive born elephants and estimated for wild caught conspecifics, which is based on a number of physical characteristics such as body size and condition, skin pigmentation, and tail hair density^70^. We extracted calving information for each female, including the dates of birth for any offspring.

### Parasite sample collection and analysis

We collected fresh, non-invasive faecal samples from elephant hosts using a species-specific, standardized method^45^. Samples were either examined from fresh or refrigerated at approx. 4–6 °C for up to five days following collection. At the same time of parasite sample collection, we recorded the dates of last deworming treatment from elephant log-books.

We carried out FECs with a special modification of the McMaster method^71^; we suspended 4.5 g of faeces in 40.5 ml saturated salt solution (NaCl, specific gravity 1.18–1.20) which we then transferred to a two-chambered McMaster counting slide and left for 5 minutes before counting. To quantitatively estimate nematode load, we carried out FECs, observing the whole area of the McMaster slides using compound microscopes with 10x magnification and 10x optical zoom. FECs were multiplied by 10 to convert counts into eggs per gram of faeces (epg). Observed ova were visually identified to the lowest possible taxonomic unit during FECs by recognition of distinctive morphological characteristics^72^. A subsample of eggs were pictured and measured using Fiji^73^, see Supplementary Fig. S1.

Faecal egg counts are widely used to reliably quantify nematode burdens in different host taxa^74^. However, no studies have yet established the exact relationship between FECs and other quantifiers, or ultimate levels of infection in elephants, for example adult worm abundance. As such, FECs only provide a quantified estimation of nematode load for elephants with several caveats. FECs only assess the presence of reproductive adult nematodes and the relative rates of egg shedding in females. FECs cannot therefore account for numbers of immature larvae, variation in female fecundity and egg output, including (for high burdens) pulsed reproduction and reproductive suppression of nematodes, nor for differences in prepatent periods between nematode species. Furthermore, the diversity of parasite species captured by faecal egg counts may be limited by the floatation solution used; a solution with a narrow specific gravity cannot capture of a broad range of parasite species, which may float at higher or lower specific densities^75^. However saturated salt solution, as used in our study, are effective in floating common helminth eggs and protozoan cysts^75^, and are widely used for such. As such, FECs should be considered as an estimation only of nematode burdens in most host systems, particularly if linear associations with burdens are yet to be confirmed through post-mortem examination, as is the case with the timber elephant population.

### Data analysis

All statistical analyses were carried out in *R* 3.3.2^76^.

#### Aggregation of nematodes across hosts

We calculated the degree of nematode aggregation within the elephant population to establish whether observable levels of infection vary across elephant hosts. We determined nematode aggregation, *κ*, as $\frac{\left({\mu }^{2},-,(\frac{\sigma }{n})\right)}{(\sigma -\mu )}$, where *μ* is mean nematode FEC, *σ* is the variance and *n* the number of elephant hosts^3^.

### Variation in nematode load with host age and sex

We applied generalized linear mixed effects models (GLMMs) to establish host-specific heterogeneity in nematode load associated with elephant age and sex. We included a negative binomial framework and log link, and untransformed FECs as our response variable, with *lme4* package^77^. We assessed the empirical mean-variance relationship of our data to verify our model structure was appropriate for a negative-binomial distribution (Fig. S3). Initially we included five fixed terms: host age (years), sex, collection season (hot, monsoon or cold), elephant camp (Kawlin, East Katha and West Katha), and a term to account for time since last anthelmintic treatment (number of days). We accounted for non-independence of repeated measures from the same hosts, or within the same year, by including two random intercepts (elephant ID and year of sample collection, both categorical terms).

Parasite infection (as indicated by FECs) may follow a polynomial trajectory with host age (e.g. age intensity curves as described by Wilson^3,14^). We therefore determined the relationship between FEC and elephant age using likelihood ratio tests (LRTs) to compare models which included age as linear (age), quadratic (age2) or cubic terms (age3), i.e. no age vs age, age vs age + age2, and age + age2 vs age + age2 + age3. We retained age in the model structure at the highest significant polynomial level. We next tested the significance of all other terms, again using LRTs to compare the model to replicates, each missing a singular fixed or random term in turn.

Finally, we tested whether any age-specific changes in infection differed between males and females by comparing our starting model to those also including interaction terms between age (all significant polynomial levels) and sex. We retained significant confounding fixed terms and significant random terms to create a final model structure, henceforth referred to as the demographic model.

### Variation in nematode load with female reproduction

We next investigated whether infection differed between mothers and nulliparous females. We restricted our dataset to females of a minimum of 13 years of age (mean age of first reproduction minus standard deviation for female timber elephants^78^). This resulted in 532 FECs from 133 females of reproductive age. Our initial model framework included host age, camp, season, days since last treatment, elephant ID and year of sample collection, as described above. We took multiple approaches to test whether infection was associated with female reproduction. Using our initial model structure, we created four models which tested for associations between nematode FECs and (1) whether females had ever reproduced by the time of sample collection or not (two-level categorical representing long-term reproductive effort), (2) whether females had produced a calf within five years of sampling (two-level categorical representing short-term reproductive effort), (3) the number of progeny produced over a female’s lifetime until sampling (continuous term representing lifetime reproductive output, maximum 10 calves), and (4) whether females were pregnant or not at the time of sampling. Timber elephant calves are dependent upon their mothers for milk until approximately five years of age. We therefore defined short-term reproduction effective effort as five years from sampling, to reflect the time following parturition that mothers directly invest in calves through lactation, and thus when calves present a direct physiological burden. Additionally, gestation in elephants can span up to approximately 22 months^44^ and may not be physically recognizable in females at the start of pregnancy. We thus restricted our dataset when testing associations between infection and state of pregnancy (model 4) to samples collected before two years prior to the last sampling date, to ensure we analysed samples from females which would have produced a calf before the end of the study period. We then used exact dates of births of calves to calculate whether females had been pregnant during dates of sampling, resulting in 276 observations from 85 females.

### Individual repeatability of infection

Finally, we assessed the repeatability of nematode infection within individual elephant hosts. We limited our data to elephants that had been sampled at least twice over the entire study period. We then applied our final demographic model structure and estimated adjusted repeatability using rptR with an overdispersion term fitted to a Poisson distribution^79^, applying non-parametric bootstrapping, and using ID number as a grouping factor. We calculated asymptotic 95% confidence intervals (CIs) from 1000 bootstrapping runs and 100 permutations.

## Supplementary information

Supplementary information.

## Data Availability

Data will be made publicly available in a depository (e.g. Dryad) upon acceptance of the manuscript.
