# Supplementary material for: Demographic and reproductive associations with nematode infection in a long-lived mammal
Source: Sci Rep. 2020 Jun 8;10:9214. doi: 10.1038/s41598-020-66075-w (PMC7280280; doi:10.1038/s41598-020-66075-w)
Supplement: Supplementary file 1 — Supplementary information. [file 41598_2020_66075_MOESM1_ESM.docx]

Demographic and reproductive associations with nematode infection in a long-lived mammal

Carly L. Lynsdale^*1^, Nay Oo Mon^2^, Diogo J. Franco dos Santos^3^, Htoo Htoo Aung^4^, U Kyaw Nyein^4^, Win Htut^4^, Dylan Childs^3^ and Virpi Lummaa^1^.

*clynsdale@gmail.com

^1^Department of Biology, University of Turku, Turku, Finland

^2^Department of Animal Science, University of Veterinary Science, Yezin, Myanmar

^3^Department of Animal and Plant Sciences, University of Sheffield, Sheffield, UK

^4^Myanma Timber Enterprise, Ministry of Natural Resources and Environmental Conservation, Yangon, Myanmar


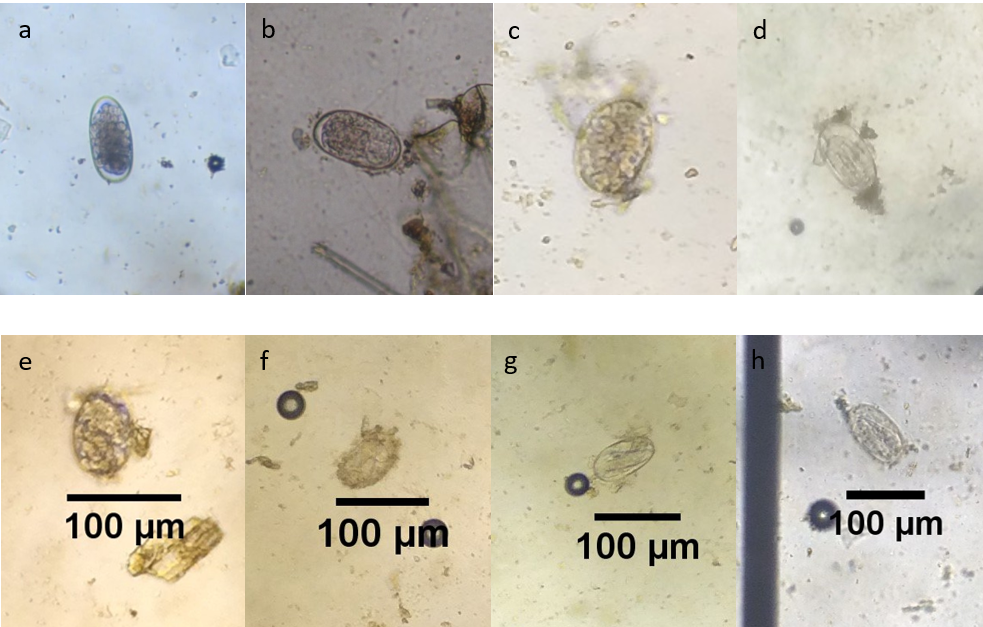
Supplementary Figure S1. Typical eggs observed during faecal egg counts of similar structure and size (e.g. S1e-h). Eggs appeared to be in different stages of development, being morulated (e.g. S1a), embryonated (S1b-c) or larvated (S1d). For a small number of eggs, faecal debris had accumulated on the shell (e.g. S1f).


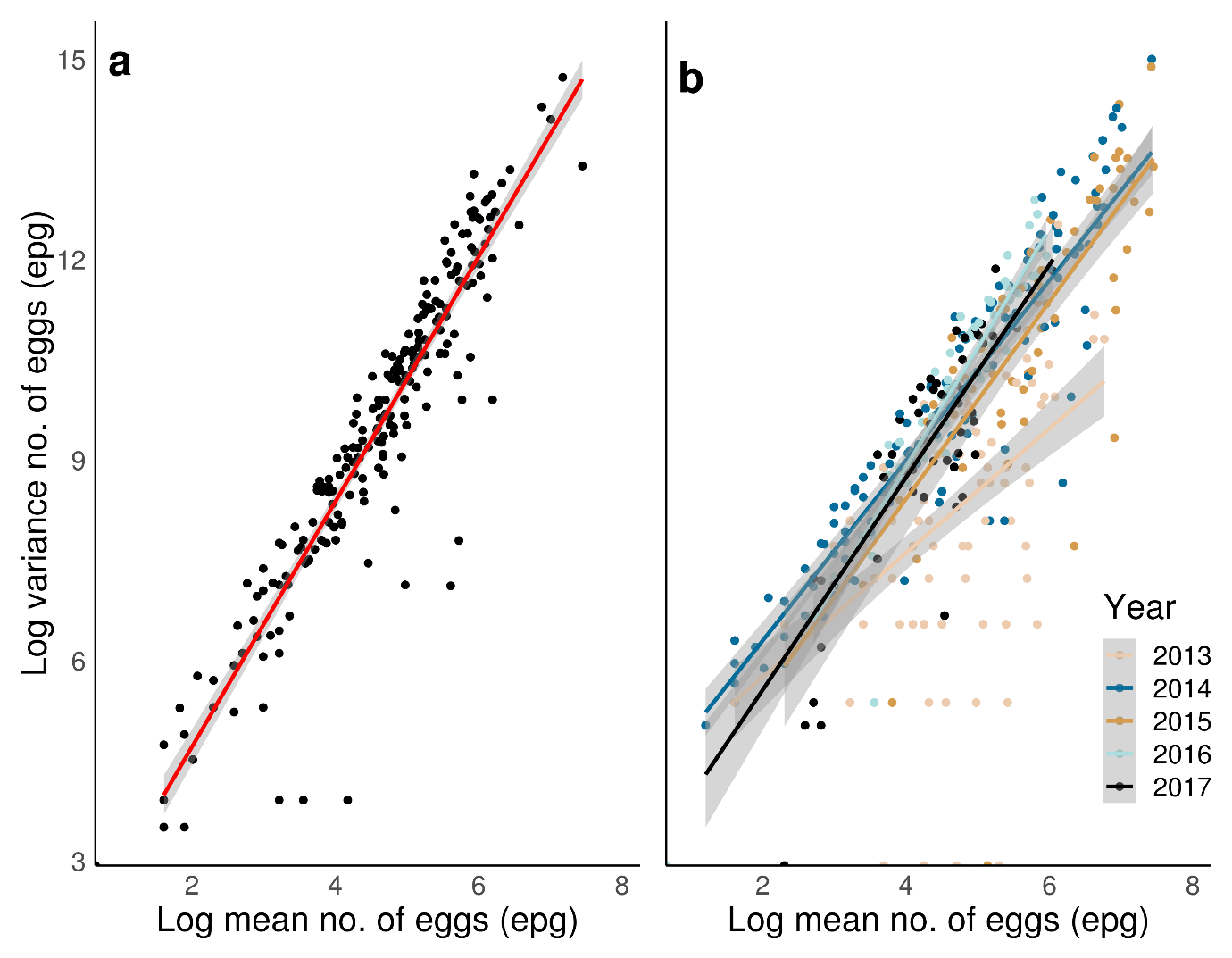
Supplementary Figure S2. The variance-to-mean ratio for overall study period (a) and year-to year (b) for the number of nematode eggs observed per elephant host, plotted on the log scale. For (a) n = 232 elephants, and for (b) n = 117 (2013), 151 (2014), 97 (2015), 48 (2016) and 66 (2017). For all measures in (a) and within grouped years in (b) all elephants were sampled 2 – 26 times. Points are raw values of the variance divided by the mean number of nematode eggs, converted to epg, for each elephant, and the solid line represents the fitted regression line for the population. For (a) adjusted R^2^ = 0.64 and for (b) adjusted R^2^ = 0.20 (2013), 0.55 (2014), 0.38 (2015), 0.54 (2016) and 0.62 (2017), calculated using the *lm* function from the ‘base’ package in R.

Supplementary Figure S3. Mean squared residuals (y-axis) vs mean fitted values (x-axis) and squared residual variance (y-axis), within equal-size bins (double log scales), for our final demography model structure. The slope of the relationship is approximately equal to the exponent of the empirical mean-variance relationship. The orange line is a fitted generalized additive model to summarise the empirical relationship and the blue line is a fitted linear model. The red line represents y = 2x.
